# Occurrence of *Listeria* spp. in Soft Cheese and Ice Cream: Effect of Probiotic *Bifidobacterium* spp. on Survival of *Listeria monocytogenes* in Soft Cheese

**DOI:** 10.3390/foods11213443

**Published:** 2022-10-30

**Authors:** Rania M. Ewida, Walaa S. Hasan, Mohamed Salem Elfaruk, Raed Reshaid Alayouni, Ahmed R. A. Hammam, Dalia G. Kamel

**Affiliations:** 1 Food Hygiene (Milk Hygiene), Faculty of Veterinary Medicine, New Valley University, Kharga 72511, Egypt; 2 College of Milk Hygiene, Veterinary Teaching Hospital, Faculty of Veterinary Medicine, Assiut University, Assiut 71526, Egypt; 3 Dairy and Food Science Department, South Dakota State University, Brookings, SD 57007, USA; 4 Medical Technology College, Nalut University, Nalut 00218, Libya; 5 Department of Food Science and Human Nutrition, College of Agriculture and Veterinary Medicine, Qassim University, Buraydah 51452, Saudi Arabia; 6 Dairy Science Department, Faculty of Agriculture, Assiut University, Assiut 71526, Egypt

**Keywords:** listeriosis, probiotic, *Bifidobacterium*, dairy products, preservatives, inhibition

## Abstract

*Listeria monocytogenes* is one of the most important emerging foodborne pathogens. The objectives of this work were to investigate the incidence of *Listeria* spp. and *L. monocytogenes* in soft cheese and ice cream in Assiut city, Egypt, and to examine the effect of some probiotic *Bifidobacterium* spp. (*Bifidobacterium breve*, *Bifidobacterium animalis*, or a mixture of the two) on the viability of *L. monocytogenes* in soft cheese. The existence of *Listeria* spp. and *L. monocytogenes* was examined in 30 samples of soft cheese and 30 samples of ice cream. Bacteriological analyses and molecular identification (using 16S rRNA gene and *hlyA* gene for *Listeria* spp. and *L. monocytogenes*, respectively) were performed on those samples. Additionally, *Bifidobacterium* spp. were incorporated in the making of soft cheese to study their inhibitory impacts on *L. monocytogenes*. Out of 60 samples of soft cheese and ice cream, 25 samples showed *Listeria* spp., while *L. monocytogenes* was found in only 2 soft cheese samples. Approximately 37% of soft cheese samples (11 out of 30) had *Listeria* spp. with about 18.0% (2 out of 11) exhibiting *L. monocytogenes*. In ice cream samples, *Listeria* spp. was presented by 47% (14 out of 30), while *L. monocytogenes* was not exhibited. Moreover, the addition of *B. animalis* to soft cheese in a concentration of 5% or combined with *B. breve* with a concentration of 2.5% for each resulted in decreasing *L. monocytogenes* efficiently during the ripening of soft cheese for 28 d. *Listeria* spp. is widely found in milk products. Probiotic bacteria, such as *Bifidobacterium* spp., can be utilized as a natural antimicrobial to preserve food and dairy products.

## 1. Introduction

*Listeria* spp. are ubiquitous and easily assimilated into the food chain. They are found in water, soil, feces, and on plant surfaces [1]. *Listeria monocytogenes* is the major species that causes foodborne diseases, and it has resulted in several outbreaks due to the consumption of raw milk or soft cheeses [2,3]. Additionally, *Listeria* can cause listeriosis in neonates, elderly, pregnant women, and immune-compromised patients, which threatens those people’s lives [4]. Listeriosis can result in slight symptoms (e.g., non-invasive febrile gastroenteritis) or severe symptoms (e.g., septicemia, meningitis, abortion) with a high chance of death [3,5]. Between 1998 and 2008, at least 25% of reported listeriosis outbreaks in the United States were caused by dairy products [6].

Ice cream and cheese are very common dairy products around the globe due to their nutritional values. There are several studies that reported the existence of *Listeria* spp. or *L. monocytogenes* in dairy products [7,8,9,10,11,12,13,14]. It was found that *L. monocytogenes* was presented in cheese samples at a ratio of 6.7% (6 out of 90 cheese samples) in Brazil, while 60 ice cream samples did not show any *L. monocytogenes* [12]. Another study reported that 8.2% of soft cheese samples (63 out of 769) had *L. monocytogenes* [8]. *L. monocytogenes* was also found at 3.5% (21 out of 603 samples) and 0.8% (2 out of 256 samples) in ice cream and soft cheese, respectively, in Chile [10].

*L. monocytogenes* can survive in extreme environmental conditions like freezing, vacuum, and ultraviolet (UV) as well as low-temperature long-time pasteurization [15,16,17]. Moreover, it can grow in a wide range of temperatures (1.0–45.0 °C), pH (4.4–9.0), and salt concentrations (up to 10%), which makes it difficult to control [18].

The application of chemical preservatives in food leads to many public health problems, such as the elevation of the chemical residues in food and increase microbial resistance, which eventually affect human health [19,20]. Today, natural biopreservative agents are utilized in food manufacture, since they do not affect consumer health, and they have many health benefits, such as increasing immune system response and protecting humans from infective microorganisms, for instance, food poisoning bacteria.

Probiotic bacteria are live microorganisms which can provide health benefits in a concentration of 5.0 to 7.0 log cfu/g [21]. Probiotic bacteria are one of the biopreservative highly used in dairy products. In Egypt, most of the soft cheeses are made using rennet only without any starter. Several studies have presented the possibility of using probiotics to inhibit growth *L. monocytogenes* in different dairy products [22,23,24,25,26]. Probiotics could have an antilisterial effect and might be utilized as additives to inhibit the growth of *L. monocytogenes* in food [27]. *Bifidobacterium* strains are some of the probiotic bacteria which have several benefits to human health, namely strengthening the immune system [28], inhibiting different entero-pathogens [29], preventing inflammatory bowel diseases [30], and reducing colon cancer risk [31,32]. Additionally, they improve lactose tolerance, calcium absorption, and vitamin synthesis while lowering serum cholesterol levels [33,34].

To the best of our knowledge, no study has presented the inhibitory effect of *Bifidobacterium* strains on the growth control of *L. monocytogenes* in soft cheese. Therefore, the objectives of this work were to investigate the incidence of *Listeria* spp. and *L. monocytogenes* in commercial soft cheese and ice cream in the city of Assiut, Egypt, and to examine the inhibitory effect of *Bifidobacterium breve*, *Bifidobacterium animalis*, or a mixture of the two on the growth control of *L. monocytogenes* in soft cheese.

## 2. Materials and Methods

### 2.1. Sample Preparations

Sixty samples of soft cheese and ice cream were obtained from various commercial markets in Assiut, Egypt, from January to June 2021. Each cheese sample was placed in a sterilized container under cooling conditions. The ice cream samples were immediately stored in iceboxes during transfer to the lab. A 25 g of each sample was prepared for analysis [35].

### 2.2. Isolation and Identification of Listeria *spp*.

The isolation and identification of *Listeria* spp. were performed, as described previously by Roberts and Greenwood [36], under aseptic conditions. A 225 mL of *Listeria* enrichment broth medium (CM0862, Oxoid, UK) containing *Listeria* oxford antimicrobic supplement (4240038, Biolife, Italy) was used to homogenize the sample and then incubated for 1–2 d at 30 °C. The abovementioned broth was then subcultured on an Oxford *Listeria* agar medium (CM0856, Oxoid, UK) adjunct with Oxoid™ *Listeria* selective supplement (SR0140, Oxoid, UK). The latter was then incubated at 30 °C for 48 h. The color of *Listeria* spp. colonies typically ranged from greyish green to brownish green with black zones of 1–3 mm diameter of aesculin hydrolysis.

Five *Listeria* spp. colonies were selected from each Petri dish of selective agar and cultivated on trypticase soy agar medium (CM0131, Oxoid, UK) supplemented with 0.6% yeast extract and subsequently placed into an incubator at 30 °C for 24 h to perform further analyses, including biochemical assays. Non-spore Gram-positive coccobacilli strains were examined for catalase, umbrella growth in motility, nitrate reduction, MR/VP, β-hemolysis production, CAMP tests (synergistic lysis of red blood cells) against *Staphylococcus aureus*, and acid formation from glucose, rhamnose, xylose, and mannitol fermentation [36,37].

**Polymerase chain reaction (PCR).** The DNA isolation of the suspected strains and the standard strain was streaked onto nutrient broth medium and then incubated overnight at 37 °C for DNA extraction by Patho Gene-spin TM DNA/RNA Extraction kit (iNtRON Biotechnology, Seongnam-Si, South Korea, ISO 9001/14001). This was performed following the instructions of the kit. The extracted DNA was stored at −20 °C [38].

The forward and reverse primer made as 907r (CCGTCAATTCCTTTGAGTTT’) and 27f (AGAGTTTGATCCTGGCTCAG) [39] were used to amplify a 900 bp region in the 16S rRNA gene for the detection of *Listeria* genus. Furthermore, another pair was created to detect *L. monocytogenes* isolates as F:GCAGTTGCAAGCGCTTGGAGTGAA and R:GCAACGTATCCTCCAGAGTGATCG were used to detect the harboring *hlyA* gene, which amplified a 456 bp fragment (Applied Biosystem, Foster City, CA, USA) [40].

We followed the same procedures as described previously in our study [41]. PCR was carried out on a total volume of 25 μL as follows: 12.5 μL of 2X PCR master mix (Green Master, Promega, Madison, WI, USA), 150 ng of the DNA template, 0.5 μM of each primer, and 25 μL Nuclease free water were thoroughly mixed in a PCR tube. The amplification occurred in Gradient Thermal Cycler (Veriti Applied Biosystem, USA), 2 min at 94 °C, then 35 cycles were run according to the following conditions: 2 min denaturation at 94 °C, annealing at 55 °C for 45 s, and extension at 72 °C for 2 min. Eventually, the mixture was kept for 7 min at 72 °C as a final extension.

PCR products were electrophoresed in 1% agarose gel (GX 040.90, Gen AGarose, L.E., Standard DNA/RNA agarose, Molecular Biology Grade, Inno–Train Diagnostic, D–61476, Kronberg/Taunus) containing ethidium bromide as 1 µL ml^−1^ electrophoresis buffer at 100 volts for 60 min. Using 100 bp DNA–ladder in (SCiE–PLAS, HU 10, 5636, Cambridge, UK). The data were recorded using high performance UV trans illuminator (UV, Inc., Leicester, UK). The images of the PCR end products were analyzed using DOC–It ^®^ LS, Image acquisition software (Biodoc Analyzer, Biometra, Germany).

### 2.3. Effect of Some Bifidobacterium *spp.* on Survival of L. monocytogenes in Soft Cheese

***Preparation of L. monocytogenes*.** The previous isolated *L. monocytogenes* were propagated in tryptic soy broth for 24 h at 37 °C. As described in a previous study [42] with some modifications, 1 mL of the culture was regularly diluted in peptone/saline solution to obtain the desired concentration and then added to the milk to yield a concentration of 10 log cfu mL^−1^.

***Preparation of Bifidobacterium spp*.** The reference strains of *Bifidobacterium breve* (ATCC 15700) and *Bifidobacterium animalis* subsp. *lactis* (CIP 105265) were obtained from the Egyptian Microbial Culture Collection (Cairo, Egypt). The strains were propagated in MRS broth (de Man, Rogosa, and Sharpe broth, CM0361, Oxoid, UK) at 37 °C for 24 h under anaerobic conditions. One milliliter of the culture was diluted using a serial method in peptone/saline solution to attain the desired inoculum level and then added to the milk to produce a 10 log cfu mL^−1^ concentration.

### 2.4. Manufacture of Soft Cheese with Bifidobacterium *spp*.

Soft cheese was manufactured from milk where 10 L of milk were pasteurized at 63 °C for 30 min and cooled to 40 °C. Then salt and rennet were added at a concentration of 15 g L^−1^ and 15 mL L^−1^, respectively [43,44]. The milk was divided into 4 aliquots; the first one was made without any additives, the second part was manufactured using *B. breve* (5%), the third part was made using *B. animalis* (5%), and the fourth part made through the addition of the two previous strains (2.5% for each strain). The treated milk was left for 45 min at 30 °C in an incubator until the curd was set, and then the whey was drained. Cheese samples were kept refrigerated at a low temperature (4 ± 2 °C) and analyzed for *L. monocytogenes* count, *Bifidobacterium* count, and pH at 0 h (after making the curd), 1, 2, 3, 7, 14, 21, and 28 d.

### 2.5. Bacteriological Examination of Soft Cheese

Under a complete septic condition, 10 g of each cheese sample were placed into a sterile mortar with sterile white sand. These samples were thoroughly ground and mixed with 90 mL of sterile 2% sodium citrate solution to obtain a dilution of 10^−1^, from which decimal serial dilutions were prepared [35,37]. Over a dry surface oxford *Listeria* selective agar (CM0856, Oxoid, UK) supplemented with *Listeria* selective supplement (SR0140, Oxoid, UK) and MRS agar (Himedia, India), 0.1 mL from every serial dilution of samples under examination was transferred and evenly spread using surface plating technique. The inoculated Oxford *Listeria* selective agar plates were incubated at 30 °C for 48 h [36], while inoculated MRS agar was incubated anaerobically at 37 °C for 7 d. The number of colonies in countable plates was enumerated and *L. monocytogenes* and *Bifidobacterium* counts per g were calculated and recorded.

### 2.6. Measurement of pH

The pH value of each sample was determined according to the standard methods [35] using a calibrated pH meter (with a buffer solution of pH 4.0 and 7.0) (Hanna, Portugal).

### 2.7. Statistical Analyses

Statistical analysis was performed using R software (R ×64−3.3.3, R Foundation for Statistical Computing) to study the effects of treatment and storage time and the interaction of those factors on the shelf-stability of soft cheese. Two-way ANOVA was performed to compare means at *p* < 0.05 using the Tukey test.

## 3. Results and Discussion

**Figure 1:**
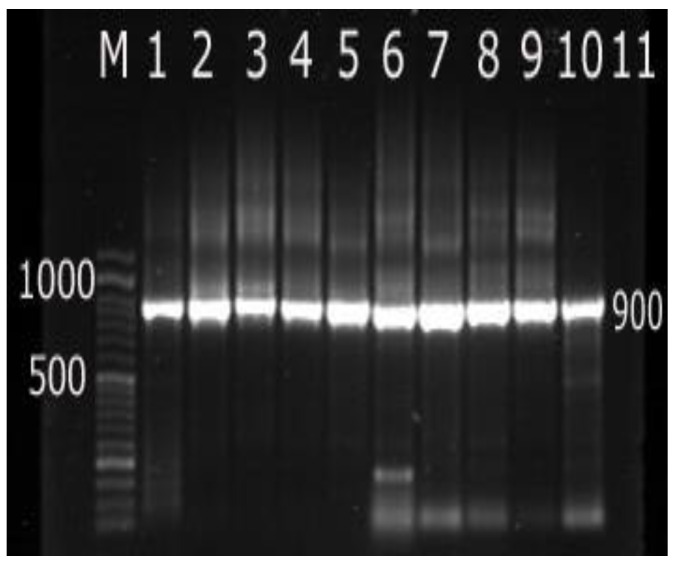
Polymerase chain reaction (PCR) products of amplified 16S rRNA of *Listeria* spp. Lane M = DNA ladder 50 bp; Lane 1–9 = positive strains with specific bands at 900 bp; Lane 10 = positive control (*L. monocytogenes* ATCC 7644); Lane 11 = negative control (*Bifidobacterium breve* ATCC 15700).

Table 1 shows the existence of *Listeria* spp. in soft cheese and ice cream samples. The incidence of *Listeria* spp. was 25 (41.66%) out of the obtained 60 samples of dairy products. Eleven samples (36.66%) were contaminated with *Listeria* spp. in soft cheese samples, while fourteen samples (46.66%) of the ice cream samples contained the same bacteria (Figure 1). These results were slightly lower than the results obtained and reported in another study [45], at which *Listeria* spp. was found in 40% of the examined cheese samples. However, the obtained data were higher than other values reported in different studies [37,46,47,48,49]. The differences in contamination of dairy products with *Listeria* spp. could be related to the properties of initial material as well as processing and environmental conditions.

**Figure 2:**
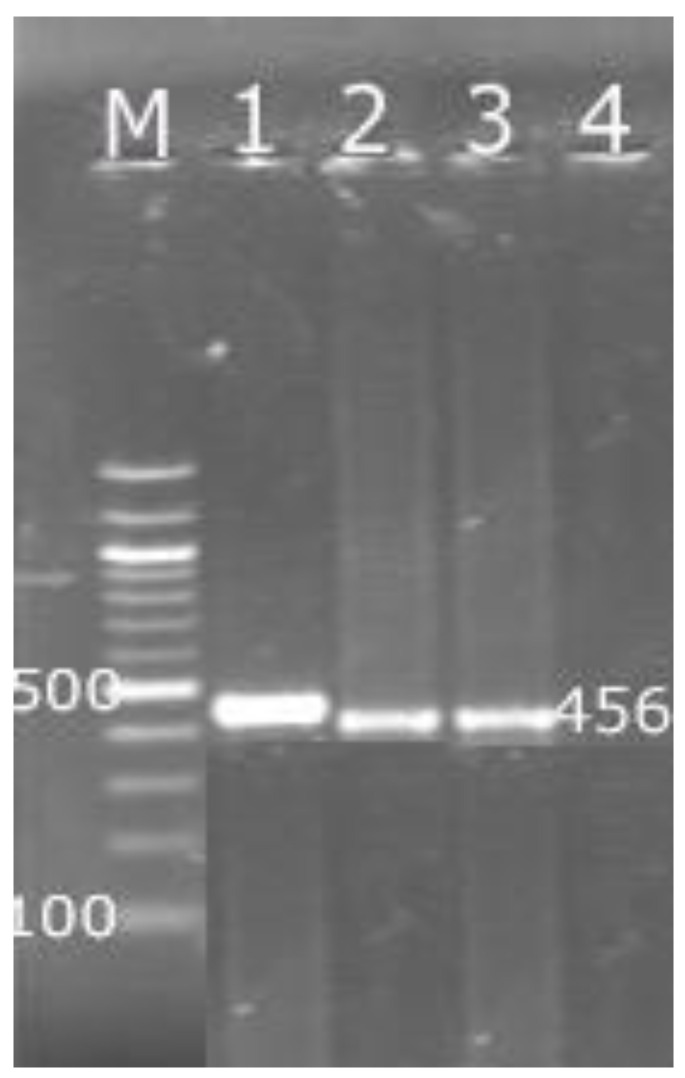
Polymerase chain reaction (PCR) products of *hlyA* fragments from *Listeria* monocytogenes. Lane M = DNA ladder 50 bp; Lane 1 and 2 = positive samples with specific bands at 456 bp; Lane 3 = positive control (*L. monocytogenes* ATCC 7644); Lane 4 = negative control.

Table 2 presents the presence of *L. monocytogenes* in cheese and ice cream samples. The frequency distribution of *L. monocytogenes* in the positively examined samples was two samples (18.18%) in the soft cheese samples (Figure 2). On the other hand, *L. monocytogenes* were not detected in the ice cream samples. The incidence values of *L. monocytogenes* in soft cheese samples is higher than the results pointed out by other researchers [47,49]. Moreover, the incidence of *L. monocytogenes* in the examined ice cream samples in this study is similar to another study [50], while the count of *L. monocytogenes* in this study is lower than the results carried out in other studies [45,46]. One study in Brazil detected *L. monocytogenes* in cheese samples at a ratio of 6.7%, and this was not found in ice cream samples [12]. Another study reported that 63 soft cheese samples out of 769 had *L. monocytogenes* [8]. *L. monocytogenes* was also found in Chile in 21 out of 603 ice cream samples and 2 out of 256 soft cheese samples [10].

The high incidence of *Listeria* spp. could be due to the use of unpasteurized milk, unsanitary production, and food storage, as *Listeria* spp., in particular, can maintain its growth rate in low salt concentration media and low temperatures (4 °C) [15,16,17,18]. As a result, the cheese could be the suitable media for the growth and multiplication of the different species of *Listeria*, including *L. monocytogenes*.

Table 3 exemplifies the inhibitory impact of *Bifidobacterium* spp. on *L. monocytogenes* in cheesemaking. The count of *L. monocytogenes* decreased gradually in the control sample until 28 d of storage at 4 °C. The count began at 10.5 log cfu g^−1^ and reached 5.3 log cfu g^−1^ by the end of the 28 d. On the other hand, the *L. monocytogenes* count decreased in the soft cheese made with *B. breve* during storage. It could not be detected in the sample at the end of storage with a reduction percentage of 100% at 28 d of storage. Moreover, the addition of *B. animalis* only or combined with *B. breve* to the soft cheese produced a high reduction rate that reached 100% after 21 d, and *L. monocytogenes* could not be isolated from soft cheese after 21 d of storage at 4 °C. In comparison between the percentage reduction of *L. monocytogenes* in soft cheese made with *B. animalis* and soft cheese made with the mix, the percentage reduction of the first treatment ranged from (13.0 to 100%), while the percentage reduction of the second treatment ranged from (1.8 to 100%). From those data, the optimum method for inhibiting the growth of *L. monocytogenes* (*p* < 0.05) is using *B. animalis* at a 5% concentration when making cheese. Many previous studies reported the effect of *Bifidobacterium* spp. against *L. monocytogenes* strain [51,52,53,54]. This is due to the ability of *Bifidobacterium* spp. to produce antibacterial peptides, such as bacteriocins, which have an inhibitory impact on the growth of *L. monocytogenes* [25,55].

Table 4 displays *Bifidobacterium* count in soft cheese made with *Bifidobacterium* spp. during storage for 28 d. Table 5 shows the pH of the cheese during storage for 28 d. As can be seen from Table 4, the *B. breve* and *B. animalis* counts gradually increased from 0 d until the end of 28 d in the soft cheese made using these bacteria and rennet. This might be due to the fact that pH did not decrease significantly (Table 5). The *Bifidobacterium* spp. increased from approximately 10.0 log cfu g^−1^ to around 11.0 log cfu g^−1^ with a slight change in pH in all treatments. Moreover, the high count of *Bifidobacterium* spp. In the finished product could provide many benefits to the health of consumers.

Strains of *Bifidobacterium* spp., such as *B. animalis* and *B. breve*, are used as adjunct cultures in several dairy products. Cultures of *Bifidobacterium* have been utilized efficiently in some cheese studies, since the pH and fat content of cheeses, as well as their buffering impact, may increase the viability of those microbes in terms of shelf-life [56]. In addition, the cheese matrix also has a role in sustaining the probiotic bacteria in low pH and bile salts present in the gastrointestinal tract of the human after cheese consumption [57]. Moreover, *Bifidobacterium* spp. has an inhibitory effect on intestinal pathogens, due to elevating the antibody production, blocking adhesion sites on the mucosal epithelium, decreasing the pH of the gut, blocking toxin receptor sites, and the secretion of antibacterial protein compounds, such as bacteriocins [58].

## 4. Conclusions

In conclusion, the results obtained in this study demonstrate that soft cheese and ice cream can be contaminated by *Listeria* spp. Traditionally, soft cheese has been viewed as the source of *L. monocytogenes* infection. Modifications of the manufacture of soft cheese through the addition of certain species of *Bifidobacterium*, especially *B. animalis*, can lead to a significant antilisterial effect.

## Figures and Tables

**Table 1:** *Listeria* spp. in the examined soft cheese and ice cream samples.

Samples	No.	No. of Contaminated Samples	
		No.	%
Soft cheese	30	11	36.66
Ice cream	30	14	46.66
Total	60	25	41.66

**Table 2:** *Listeria monocytogenes* recovered from the examined soft cheese and ice cream samples.

Samples	No.	No. of Contaminated Samples	
		No.	%
Soft cheese	11	2	18.18
Ice cream	14	-	0
Total	25	2	8.00

**Table 3:** Count (log cfu g^−1^) and percentage reduction of *L. monocytogenes* inoculated in manufactured soft cheese with *Bifidobacterium* spp. stored at 4 ± 2 °C.

Storage Time (d)	Treatments ^1^							SEM	*p*-Value ^2^		
	Rennet Cheese	*B. breve* Cheese		*B. animalis* Cheese		Mixed Culture Cheese					
	Count	Count	Red. %	Count	Red. %	Count	Red. %		Trt	Time	Trt × Time
0	10.48	10.31	1.6	9.12	13.0	10.29	1.8	0.16			
1	11.30	8.77	22.4	5.11	54.8	5.31	53.0	0.77			
2	9.16	6.46	29.5	4.05	55.8	4.78	47.8	0.59			
3	7.39	4.25	42.5	3.08	58.3	3.53	52.2	0.51			
7	6.23	3.66	41.3	2.92	53.1	2.99	52.0	0.41			
14	5.47	3.38	38.2	2.56	53.2	2.68	51.0	0.35			
21	5.23	3.04	41.9	0.00	100	0.00	100	0.66			
28	5.30	0.00	100	0.00	100	0.00	100	0.69			
SEM	0.47	0.65		0.57		0.64					
Mean	7.57 ^a^	4.98 ^b^		3.35 ^d^		3.69 ^c^			<0.05	<0.05	<0.05

^a–d^ Means in the same row not sharing a common superscript are different at *p* < 0.05. ^1^ Treatments: Rennet cheese = Soft cheese made with rennet only; *B. breve* cheese = Soft cheese made with 5% *B. breve*; *B. animalis* cheese = Soft cheese made with 5% *B. animalis*; Mixed culture cheese = Soft cheese made with 2.5% *B. breve* and 2.5% *B. animalis*. ^2^ *p*-values for the effect of treatment (Trt), Time, and Treatment × Time.

**Table 4:** Count of *Bifidobacterium* spp. (log cfu g^−1^) in manufactured soft cheese stored at 4 ± 2 °C.

Storage Time (d)	Treatments ^1^				SEM	*p*-Value ^2^		
	Rennet Cheese	*B. breve* Cheese	*B. animalis* Cheese	Mixed Culture Cheese		Trt	Time	Trt × Time
0	ND **^3^**	10.64	9.98	10.55	1.36			
1	ND	10.70	10.50	10.83	1.39			
2	ND	10.97	10.72	11.11	1.43			
3	ND	11.11	11.11	11.32	1.46			
7	ND	12.50	12.78	12.81	1.66			
14	ND	15.22	14.83	14.76	1.95			
21	ND	15.63	16.30	16.70	2.12			
28	ND	16.53	15.18	16.01	2.08			
SEM	0.00	0.48	0.48	0.48				
Mean	0.00	12.91 ^b^	12.67 ^c^	13.01 ^a^		<0.05	<0.05	<0.05

^a–c^ Means in the same row not sharing a common superscript are different at *p* < 0.05. ^1^ Treatments: Rennet cheese = Soft cheese made with rennet only; *B. breve* cheese = Soft cheese made with 5% *B. breve*; *B. animalis* cheese = Soft cheese made with 5% *B. animalis*; Mixed culture cheese = Soft cheese made with 2.5% *B. breve* and 2.5% *B. animalis*. ^2^ *p*-values for the effect of treatment (Trt), Time, and Treatment × Time. ^3^ ND= not detected.

**Table 5:** The pH of manufactured soft cheese with *Bifidobacterium* spp. stored at 4 ± 2 °C.

Storage Time (d)	Treatments ^1^				SEM	*p*-Value ^2^		
	Rennet Cheese	*B. breve* Cheese	*B. animalis* Cheese	Mixed Culture Cheese		Trt	Time	Trt × Time
0	6.50	6.20	5.70	5.76	0.10			
1	6.35	6.13	5.51	5.66	0.10			
2	5.90	5.77	5.14	5.41	0.09			
3	6.42	5.80	5.30	5.32	0.14			
7	6.43	6.38	5.75	5.89	0.09			
14	6.46	6.40	5.70	5.75	0.11			
21	6.48	6.42	5.69	5.72	0.11			
28	6.50	6.45	5.65	5.69	0.12			
SEM	0.04	0.05	0.04	0.04				
Mean	6.38 ^a^	6.19 ^b^	5.55 ^d^	5.65 ^c^		<0.05	<0.05	<0.05

^a–d^ Means in the same row not sharing a common superscript are different at *p* < 0.05. ^1^ Treatments: Rennet cheese = Soft cheese made with rennet only; *B. breve* cheese = Soft cheese made with 5% *B. breve*; *B. animalis* cheese = Soft cheese made with 5% *B. animalis*; Mixed culture cheese = Soft cheese made with 2.5% *B. breve* and 2.5% *B. animalis*. ^2^ *p*-values for the effect of treatment (Trt), Time, and Treatment × Time.

## Data Availability

Data sharing not applicable.
